# A Medical Woman to Her Sex

**Published:** 1913-11-22

**Authors:** 


### MATERNITY AND MOTHERING.

### A Medical Woman to Her Sex.

In respect of problems of maternity and motherhood changes are imminent, and we are confident that the great progress of the last thirty or forty years in the sciences of gynæcology, obstetrics, and pædiatrics will be as nothing compared with future advances and with the jealous care which the State will ultimately exercise of its children and their mothers.

This seems to us to be as reasonably certain as anything prophetic concerning mundane affairs can possibly be; but we feel equally sure that false steps will from time to time be taken in the onward march, and that they will have to be retraced. To avoid such false steps will never be easy, and if there is one thing which will render them inevitable it is to be in too great a hurry for reform. Every suggested remedy for the social evils which admittedly exist and call for redress must be most carefully examined and discussed in all its bearings before being adopted; and pressing though some of those evils are, to hurry slowly in combating them will, we hold, be the surest and, in the end, the quickest way of overcoming and obliterating them.

It is one of the stock arguments of the supporters of woman suffrage that no Parliament of men can understand or rectify the disabilities of women, and that only when women get votes and are elected to the House will any real social progress be made. This is not the place to argue for or against woman suffrage; we merely take cognisance of this argument because the suffrage agitation is one symptom of a more comprehensive movement known as "feminism." We should be sorry to attach any corporate responsibility to the feminists for the excesses of the advanced "suffragettes." But these tokens of impatience with avoidable ills in the commonwealth are symptomatic of the feminine outlook and of the spread of education among the female sex. Men recognise the imperfections of much of the social system, and one by one they eliminate to their best ability the grosser faults; but the process is too slow for the vaulting ambition of the modern feminist leader, who is all for those short-cuts which the riper political experience of man teaches him are often the longest way home.

### Woman, Marriage, and Motherhood.

It is, however, very important that a clear conception should be attained of what those grievances and disabilities are under which maternity and motherhood especially suffer, before entering on any discussion of the reforms necessary and advisable. In a recent book by a medical woman * will be found a frank statement of the position as a trained worker sees it, together with an outline of the manner in which, in her view, the old order can best be changed to a new one. One by one she reviews the factors which interfere most with the function of motherhood, such as the factory system, sweated labour, alcohol, prostitution, degeneracy, crime, and so forth, and the problems of infant mortality also receive full attention.

Such a survey of the problems which the present generation is set to solve is, needless to say, a task which is full of difficulties. To guard against overstatement, to distinguish between the striking, but exceptional, case, and the tame, but ordinary, one, to see all sides of large questions, these require unusual balance, brain, and judgment. Broadly speaking, Mrs. Chesser has succeeded very fairly well in her endeavour to present her case in a form in which it can be easily assimilated by the general public. She speaks plainly and frankly on subjects about which there is, as a rule, too much reticence and mystification. She is, in the phrase of the day, always "on the side of the angels." But, unfortunately, she commits herself to a great many statements the truth of which is highly doubtful, and to others which are certainly not true at all. These statements are chiefly on matters of opinion, and no doubt she is quite sincere in making them. The mistake she makes is in enunciating her views with a pontifical assurance such as would lead the unwary to regard them as ascertained and admitted facts. To put it brutally, Mrs. Chesser has developed a habit of twaddling which mars her book very seriously.

An excellent example of this confusion of thought occurs on pages 10 and 11. Maternity, she states, evolved into motherhood "with the appearance of the first human mother upon earth." The meaning of this sentence depends on the meanings attached by the author to the words maternity, motherhood, and human. But the context shows that Mrs. Chesser believes that the human race has been slowly evolved out of some non-human ancestry; and she proceeds to declare that the primitive mother would abandon her child in the presence of danger "without any sense of loss or remorse." What possible warrant she has for such a generalisation we cannot imagine, for it is directly negatived by our knowledge of the lower animals of to-day. Self-sacrifice in defence of offspring she speaks of as a solely human attribute, and, indeed, the whole passage abounds in loose writing and careless thinking. A little further on she appears to commit herself to the notion that the offspring of "arranged" marriages are defective and degenerate. If this were literally true the French nation (and a good many others) would have been quite extinct long ago; but it is not true, and, indeed, it is very questionable whether the children of such marriages on the French system are not actually eugenically superior to those born of parents who have been allowed to marry solely at the bidding of their own untutored inclinations.

Nor are Mrs. Chesser's statistics beyond criticism.

* *Women, Marriage, and Motherhood.* By Elizabeth Sloan Chesser, M.B. Cassell and Co. 6s. net.

On one and the same page she puts the present infant mortality of England and Wales at 105 and at 120 per thousand per annum, both figures clearly cannot be correct. Elsewhere she says the income-tax abatement allowed for children under sixteen costs the Treasury five to six million pounds a year. Now the abatement amounts to seven-and-six per child, and is allowed only to parents whose total joint income is under £500. According to Mrs. Chesser's figures, therefore, we are called upon to believe that income-tax payers in the United Kingdom whose income falls under the limit mentioned possess between them about fifteen million children, a palpable absurdity which merely shows that Mrs. Chesser's original statement is incorrect. Several other instances of the misuse of statistics could be quoted, but we pass on to a glaring confusion on the subject of heredity. We are told that "the athlete does not transmit his biceps" to his children, because "acquired characteristics have no influence on the germ plasm." To begin with, a biceps (or any other muscle) is not an acquired characteristic, but an inherited one, and in point of actual fact the athlete does tend to beget athletic children, just as the mathematician tends to hand on the exceptional ability in mathematical studies which he himself got from some previous ancestor. In the very next sentence after her acceptance of the Weissmann doctrine (not, by the way, now so universally acknowledged as Mrs. Chesser seems to think) she calmly discusses the effects of alcoholism, syphilis, and other diseases in parents on the health and vitality of their offspring.

The keynote of much of the violence of the feministic movement is the belief that men do not desire to improve the organisation of society, and cannot be trusted when they say they do. Mrs. Chesser, we are glad to note, does not harbour this delusion, nor does she entertain the fallacy that the sole basis of instantaneous reform consists in "votes for women." She proposes, indeed, an alternative scheme designed to afford women more political influence than they at present possess, without actually admitting them to Parliament or the parliamentary franchise. This scheme is somewhat complicated, and we question whether it would prove workable, but it is certainly ingenious. Notwithstanding the weak points in matters of detail, some of which we have felt obliged to dwell upon, in the main we agree with many of her propositions, though not by any means with all. We think her book will do good by calling the attention of the laity to remediable evils which the medical profession and institutional workers have long perceived and laboured, not unsuccessfully, to palliate. We commend it to all mothers and fathers, and to all citizens of either sex who possess, or desire to possess, the municipal or parliamentary franchise.

